# Extensive infectious myelitis post bariatric surgery

**DOI:** 10.1186/s12879-015-0897-9

**Published:** 2015-04-14

**Authors:** Elie Haddad, Carmen Joukhadar, Nabil Chehata, Roy Nasnas, Jacques Choucair

**Affiliations:** Infectious diseases department, Faculty of medicine, Saint-Joseph University, Hotel Dieu de France Hospital, Medical Sciences and Nursing Campus, Faculty of Medicine, Damascus Street, B.P. 11-5076, Riad El Solh, Beirut, 1107-2180 Lebanon; Infectious diseases department, Hotel Dieu de France Hospital, Alfred Naccache Boulevard, Achrafieh, Beirut, BP 16-6830 Lebanon

**Keywords:** Infectious myelitis, Cerebral abscess, Spinal abscess, Abdominal abscess, Bariatric surgery, Antibiotherapy, Abdominal fistula

## Abstract

**Background:**

Inflammatory myelopathy is an inflammatory neurological disorder of the spinal cord (myelopathy). It occurs in 1 (severe) to 8 (mild) cases/million per year. It is often referred to in the literature as “transverse myelitis” or “acute transverse myelitis”. Myelopathy and by extension myelitis, can present as pyramidal (motor), sensory, and/or autonomic dysfunction to varying degrees. Symptoms typically develop over hours to days and worsen over days to weeks. Sensory symptoms usually present as paresthesia ascending from the feet with or without back pain at or near the level of the myelitis. A cervical level focal myelitis can present as sensory symptoms restricted to the feet without ascending extension. Motor symptoms often include weakness that preferentially affects the flexors of the legs and the extensors of the arms (pyramidal distribution of weakness) and can include sphincter dysfunction.

**Case presentation:**

This is the case of a 55 years old female patient who develops sudden onset abdominal abscess one year after bariatric surgery that was complicated by an extensive infectious myelitis and cerebral abscesses without any cerebral symptoms. She received adequate antibiotherapy treatment with good evolution.

**Conclusions:**

This case is among the first in the medical literature that has occurred one year after bariatric surgery complicated by an abdominal and cerebral abscesses, and extensive infectious myelitis.

We discussed all types of myelitis including, the autoimmune and the infectious origin. We showed the progressive evolution by showing MRI sequences. We emphasized about the importance of rapid initiation of the antibiotherapy as well as adding glucocorticoids.

## Background

Inflammatory myelopathy is an inflammatory neurological disorder of the spinal cord (myelopathy) [1]. It occurs in 1 (severe) to 8 (mild) cases/million per year. It is often referred to in the literature as “transverse myelitis”or “acute transverse myelitis”. Myelopathy and by extension myelitis, can present as pyramidal (motor), sensory, and/or autonomic dysfunction to varying degrees. Symptoms typically develop over hours to days and worsen over days to weeks. Sensory symptoms usually present as paresthesia ascending from the feet with or without back pain at or near the level of the myelitis. A cervical level focal myelitis can present as sensory symptoms restricted to the feet without ascending extension. Motor symptoms often include weakness that preferentially affects the flexors of the legs and the extensors of the arms (pyramidal distribution of weakness) and can include sphincter dysfunction (Timothy W. [2]).

## Case presentation

This is a case of a 50 years old female patient who is known to be hypertensive, dyslipidemic, obese (BMI: 31.88-Height: 168 cm Weight: 90 kg), and operated of sleeve gastrectomy in 2012.

On the morning of 13/02/2013, the patient noted a near syncope episode. She was transferred to the emergency department for a fever of 39.2°C, a microcytic anemia (Hb: 7 g/dl) with elevated inflammatory markers CRP 239 mg/l and ESR 90 mm and admitted for care and investigation.

Her physical exam showed lower limbs weakness at 4/5 proximally and distally (possible movement against gravity with moderate resistance). She was put on IV meropenem after blood and urine cultures were taken. The gram color was negative so were the cultures after 48 hours.

On the following morning she started complaining of paraparesia. Her physical examination showed a motor deficit of the lower limbs at 1/5 (visible contraction without any movement) with total anesthesia in all modalities (proprioceptive and thermo-algic). A D6 level was also noted.

**Figure 1:**
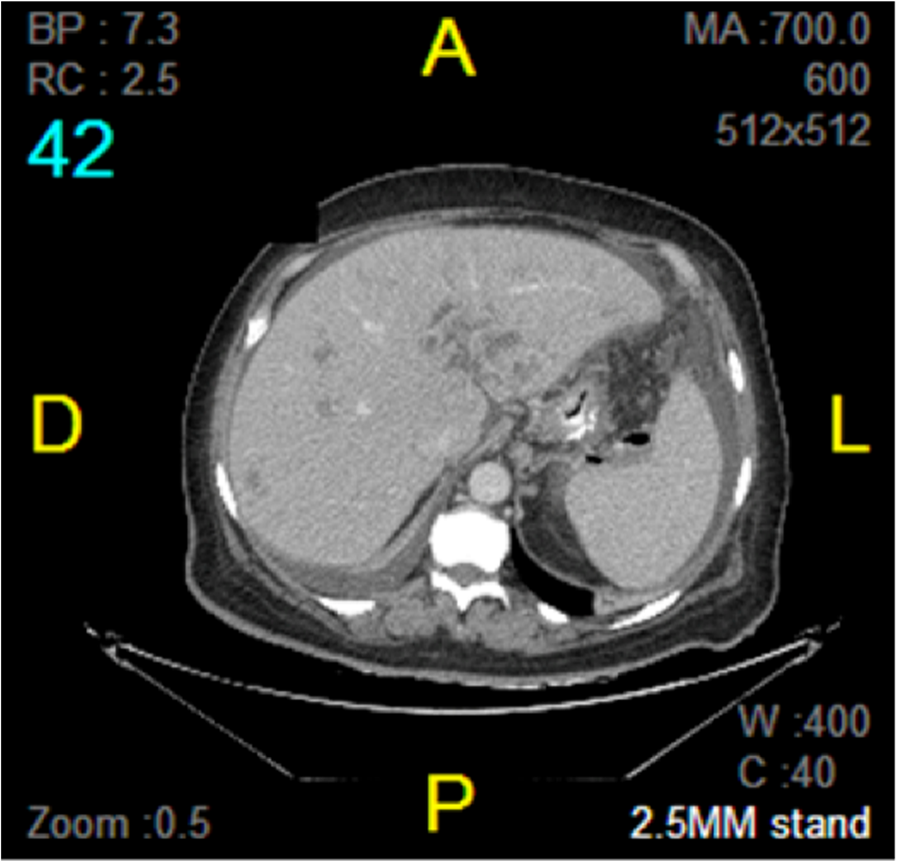
There is a collection of 42 × 15 mm opposite to the splenic hilum, which seems to communicate with the digestive tract at the proximal part of the gastro-esophago-jejunal anastomosis, suggestive of infected fistula. The gas bubbles and the presence of oral contrast seen in the collection are suggestive of fistula with the digestive tract.

An abdomino-pelvic computer tomography (CT) scan showed a complete thrombosis in the lower part of the mesenteric and the splenic veins extending to the portal bifurcation and the intrahepatic portal branches. It associated an important infiltration of the peritoneal and peri-pancreatic fat, and a mean abundance of free peri-hepatic, splenic and peri-pelvic ascites. There was a collection of 42 × 15 mm near the splenic hilum, containing a trace of contrast and a small two-fluid level. This collection seemed to communicate with the digestive tract at the gastro-esophago-jejunal anastomosis in its proximal part, suggestive of infected fistula (Figure 1).

IV heparin was added to her medications.

**Figure 2:**
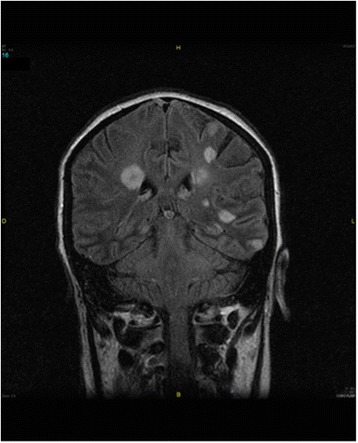
Presence of multiple small enhancing lesions scattered rosette in the brain parenchyma and cerebellar predominant in regions of the basal ganglia and the cerebral cortex, in favor of small abscesses formations.
- An extensive transverse infectious myelitis Figure 3.

An urgent brain and cervico-dorso-lombar MRI (magnetic resonance imaging) were performed and showed:

- Small brain abscesses formation Figure 2.

**Figure 3:**
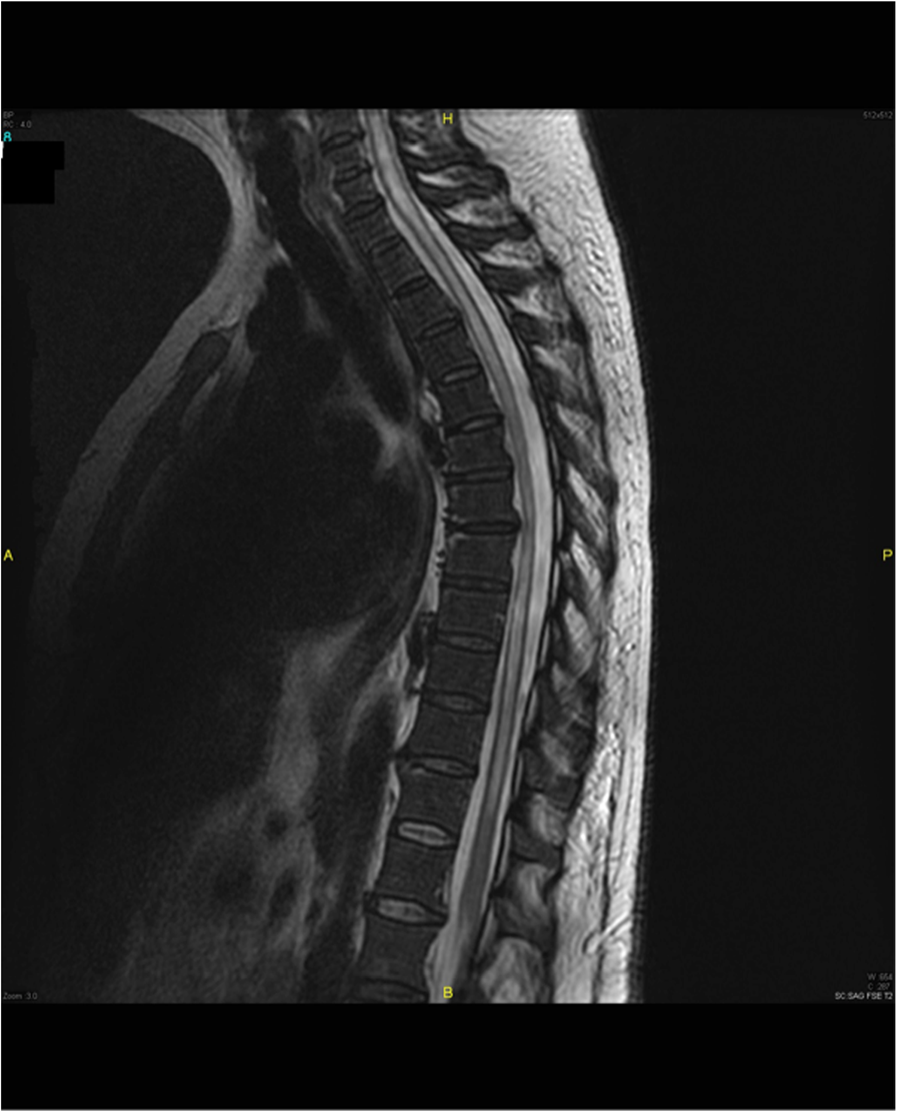
An abnormal moderately hyperintense on T2, hypointense T1, central intramedullary signal interesting almost all the cervical and dorsal spine respecting the terminal cone, and showing a contrast-enhanced periphery including a possible small necrotic remodeling over D7 and D8 with surrounding edema. The appearance is consistent with extensive myelitis, possibly infectious.

In front of these findings a lumbar puncture was performed revealing 430 WBC (92% neutrophils, 7% lymphocytes, and 1% mesothelial cells) 10 RBC with hypoglycorachia and hyperproteinorachia in favor of an inflammatory liquid with polymorphic predominance of polynucleated elements. The objective of this technique was to try to isolate a bacterium. The CSF culture returned sterile and PCR BK was negative.

An electromyogram was normal.

After this result vancomycin was added to the treatment regimen.

### Initial evaluation

Clinical presentation, physical examination, and history are important diagnostic tools in assessing an acute myelopathy as the pattern of functional loss can help to determine the location of the lesion. When an acute myelopathy is suspected, a thorough neurological evaluation will help determine the region of the spinal cord affected and then the next diagnostic step is to evaluate for a compressive or structural etiology (Timothy W. [2]).

Magnetic resonance imaging (MRI) of the complete spinal axis is mandatory in any patient with myelopathic features to exclude structural lesions, particularly those amenable to emergent neurosurgical intervention [3].

Once neuroimaging has excluded a compressive etiology, a lumbar puncture (LP) is indicated to determine if there are signs of inflammation within the cerebrospinal fluid (CSF). If the CSF is non-inflammatory, then vascular, toxic/metabolic, neurodegenerative, or neoplastic myelopathies become much more likely and the subsequent work-up should focus on these etiologies. If the CSF shows signs of inflammation (pleocytosis, elevated protein concentration, oligoclonal bands, or elevated IgG index), then the subsequent work-up should focus on demyelinating, infectious, or other inflammatory causes of an acute myelitis, as the differential diagnosis is broad [2].

### Is the myelitis caused by an infection?

However, for elucidating the etiology of infectious myelitis, CSF studies are essential. CSF studies include cell count with differential protein and glucose concentrations. In addition, measurements of intrathecal immunoglobulin production with oligoclonal bands (OCBs) and an immunoglobulin G index or synthesis rate are very helpful when trying to determine the presence and etiology of myelitis.

An elevated protein concentration is the most common CSF abnormality in patients with spinal cord disease and is present in approximately 50% of patients with transverse myelitis [4]. A low CSF glucose concentration is defined as less than 60% of serum glucose and generally suggests fungal, bacterial, or mycobacterial infection, especially when associated with an elevated CSF white blood cell count. An isolated low CSF glucose concentration can occur in neurosarcoidosis, leptomeningeal carcinomatosis, subarachnoid hemorrhage, and even systemic lupus erythematosus (SLE) with CNS involvement [5]. The white blood cell differential can be very helpful as well. The presence of eosinophils can suggest parasitic or fungal infection, the presence of foreign material (such as surgical hardware following a spinal operation) or possibly neuromyelitis optica (NMO) (Timothy W. [2]).

The most frequent pathogens involved in transverse myelitis include *Treponema palidum, Mycobacerium tuberculosis, Borrelia burgdorferi, Campylobacter jejuni, Acinetobacter baumanii, Coxiella burnetii, Bartonella henselae, Chlamydia psittaci, Leprospira, Chlamydia pneunoniae, Legionella pneumonia, Orientia tsutsugamushi (scrub typhus), Salmonella paratyphi B, Brucellosis melitensis, Group A and B streptococci* [3].

Intramedullary abscess may also complicate congenital dermal sinuses or bacterial endocarditis. So a cardiac echography should be performed to exclude endocarditis.

In our case, a PPD, HIV serology, wright and widal all returned negative. A cardiac echography was performed and ruled out any sign for endocarditis.

### Acute management

Successful treatment of infections of the nervous system depends on rapid and accurate diagnosis. The time course of symptom progression may suggest specific pathogens. In general, symptoms of less than 2 day’s duration bespeak bacterial processes. The clinical syndrome and associated extra neural infection sites favor certain organisms [6].

Empiric corticosteroids may be of benefit in bacterial [7] and in tuberculous meningitis [8]. For bacterial meningitis, the typical dosing is a four-day regimen of dexamethasone (0.6 mg/kg daily) started before or with the first dose of antibiotics [7].

Our patient had developed an abdominal abscess with fistula. The bacteria implicated in her myelitis are probably an enterobacteriaceae or an enterococcus. The CSF culture was negative probably because we already initiated the meropenem before the lumbar puncture was performed.

**Figure 4:**
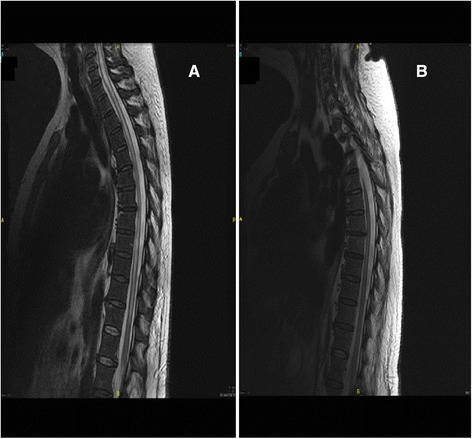
Comparison between the first MRI (figure **A**) and 2 months late (figure **B**).

A follow-up brain and cervico-dorsal MRI after 2 months of IV treatment (IV meropenem and vancomycine) showed: Figure 4.

- Net decrease of small contrast enhancing lesions scattered with persistence of nodular contrast right frontobasal, left upper front, in the region of the left and right thalamic lenticular ventricular junction, compared with small abscess formations in the process healing.
- We found a great reduction in the T2 signal abnormalities of the spinal, cervical, and thoracolumbar intramedullary spine signal. Virtual disappearance of intramedullary signal abnormality in the cervical spinal and back to a normal size.

1 year after the myelitis and physical rehabilitation, her physical exam shows motility at 3/5 (active movement against gravity) over both her lower limbs. The sensitivity over her lower limbs is recovering but poorly over the feet. Her sphincteral dysfunction is still persistent.

### Is the myelitis caused by a demyelinating or systemic autoimmune disease

The differential diagnosis with the infectious myelitis is one caused by autoimmune disease. A thorough workup was conducted eliminating all autoimmune diseases. The antinuclear antibodies, antibodies to extractable nuclear antigen, rheumatoid factor, antiphospholipid antibodies, and anti-neutrophil cytoplasmic antibodies (ANCA) are all negative [9].

CSF and blood angiotensin-converting enzyme levels are normal.

## Conclusions

Myelitis is recognized as a clinical syndrome associated with multiple etiologies [1]. As such, a detailed clinical history and physical examination are critically important. This case is among the first in the literature to occur one year after bariatric surgery. Determining the etiology of transverse myelitis can be challenging, but with effective interpretation of clinical signs and symptoms, neuroimaging, serological and CSF studies, the cause of the myelitis and the treatment course can be guided to provide the patient with the optimal chance for a good outcome. In our case, it started with an abdominal abscess from a fistula originating from the proximal part of the gastro-esophago-jejunal anastomosis. A bacterial translocation to the cervico-thoraco-lumbar spinal cord was the trigger to the transverse myelitis complicated by several brain abscesses. Rapid antibiotherapy onset with corticoids is the key factor for effective treatment.

## Consent

Written informed consent was obtained from the patient for publication of this case report and any accompanying images. A copy of the written consent is available for review by the Editor of this journal.
